# Characterization of the expressed RNA variants from young patients with critical and non-critical SARS-CoV-2 infection

**DOI:** 10.1186/s43042-022-00327-4

**Published:** 2022-08-03

**Authors:** Javan Okendo

**Affiliations:** Systems and Chemical Biology Division, Department of Integrative Biomedical Sciences, Institute of Infectious Disease and Molecular Medicine, Faculty of Health Sciences, University of Cape Town, Anzio Road Observatory, Cape Town, 7925 South Africa

## Abstract

**Background:**

Since the COVID-19 outbreak emerged, severe acute respiratory syndrome coronavirus *2* (SARS-CoV-2) has continuously evolved into variants with underlying mutations associated with increased transmissibility, potential escape from neutralizing antibodies, and disease severity. Although intensive research is ongoing worldwide to understand the emergence of SARS-CoV-2 variants, there is a lack of information on what constitutes the expressed RNA variants in critical and non-critical comorbidity-free young patients. The study sought  to characterize the expressed RNA variants from young patients with critical and non-critical forms of SARS-CoV-2 infection.

**Methodology:**

The bulk ribonucleic acid (RNA) sequencing data with the identifier GSE172114 were downloaded from the Gene Expression Omnibus (GEO) database. The study participants were divided into critical, *n* = 46, and non-critical, *n* = 23. FastQC version 0.11.9 and Cutadapt version 3.7 were used to assess the read quality and perform adapter trimming, respectively. Spliced Transcripts *Alignment* to a Reference (STAR) version 2.7.10a was used to align reads to the human (hg38) reference genome. Genome Analysis Tool Kit (GATK) best practice was followed to call variants using the rnavar pipeline, part of the nf-core pipelines.

**Results:**

Our research demonstrates that critical and non-critical SARS-CoV-2-infected individuals are characterized by a unique set of expressed RNA variants. The expressed gene variants are enriched on the innate immune response, specifically neutrophil-mediated immune response. On the other hand, the expressed gene variants are involved in both innate and cellular immune responses.

**Conclusion:**

Deeply phenotyped comorbidity-free young patients with critical and non-critical SARS-CoV-2 infection are characterized by a unique set of expressed RNA variants. The findings in this study can inform the patient classification process in health facilities globally when admitting young patients infected with SARS-CoV-2.

## Introduction

Severe acute respiratory syndrome coronavirus 2 (SARS-CoV-2) infections remain a global public health challenge [1]. Coronavirus disease 2019 (COVID-19) spreads s from person to person through direct contact or infected surfaces [2]. When SARS-CoV-2 is inhaled, it enters the human host cells via angiotensin-converting enzyme 2 (ACE2) receptors [3]. Once the virus enters the human cells, it starts replicating, leading to population expansion within the cells [3]. While in the cells, it induces the local immune cells to begin producing cytokines and chemokines, resulting in the attraction of other immune cells in the lung, which causes excessive tissue damage [4]. A growing body of evidence indicates that the SARS-CoV-2 virus is not confined to the human lungs [1]. Still, it also affects the other body organs, such as the kidney, where it causes acute kidney injury (AKI) [1, 5]. In other individuals infected with SARS-CoV-2, neurological, cardiovascular, and intestinal malfunctions have also been reported [6].

SARS-CoV-2 variant analysis has been used to identify and track the spread of SARS-CoV-2 variants of concern globally [7]. It is also understood that there is a difference in the SARS-CoV-2 prevalence in different regions, which is made possible by genome sequencing and analysis [8]. The frequency of SARS-CoV-2 reinfection in our population has been used in identifying SARS-CoV-2 variants in different parts of the world, as demonstrated by Tillett et al. [9]. Interestingly, what constitutes immune response in a broad spectrum of SARS-CoV-2 infection in our population remains an active area of research. The inherent mutational ability of SARS-CoV-2 has led to multiple variants classified into four groups: variants of concern (VOC), variants of interest (VOI), variants being monitored (VBM), and variants of high consequence (VOHC) (www.cdc.gov). The SARS-CoV-2 variants are further classified by the use of the letters of the Greek alphabet, e.g., Alpha, Beta, Delta Gamma, Iota, Kappa, Lambda, Omicron, etc., for easy-to-say labeling (www.who.int). Currently, three VBMs (Alpha-B.1.1.7, Beta-B.1.351, and Gamma-P.1) and two VOCs (Delta-B.617.2 including AY sub-lineages and Omicron-B.1.1.529 including BA lineages) are in circulation worldwide (www.cdc.gov). The Omicron variant has predominated over other variants globally [10].

Understanding the expressed RNA variants in critical and non-critical individuals infected with the SARS-CoV-2 virus will provide fundamental answers to the poorly understood SARS-CoV-2 pathogenesis. The differential manifestation of clinical features of SARS-CoV-2 will become clearer in our population and how to manage this pandemic. Studies have been conducted to characterize the SARS-CoV-2 variant using SARS-CoV-2 whole genome sequences, which have aided the identification of single nucleotide polymorphisms, insertions and deletions, and structural variants [11]. Structural bioinformatics has also been used to identify the effects of SARS-CoV-2 mutations on the native structure of the S-protein of SARS-CoV-2 by studying the D614G mutation [12]. In another related study, the effect of SARS-CoV-2 in the human host was investigated, and it was demonstrated that SARS-CoV-2 infection increased the expression of angiotensin-converting enzyme 2 (ACE2) in the pancreatic islet cells in diabetic donors [13]. This study used the bulk RNAseq variant calling approach to study the expressed variants from individuals with critical and non-critical SARS-CoV-2 infection. The findings in this study will add another layer of information that can inform the development of new modalities.

## Materials and methods

### Study samples description

In this study, I analyzed sixty-nine bulk RNA sequencing data from the Carapito et al., 2022 study [14]. The data I analyzed are available on Sequence Read Archive (SRA) with accession number PRJNA722046. The study participants were divided into two groups, non-critical, *n* = 23, and critical, *n* = 46. The individuals admitted to the intensive care unit (ICU) with acute respiratory distress syndrome (ARDS) were considered critical, and those in non-critical care wards with oxygen supplements were considered non-critical. Further details on the bulk RNA sample collection and preparation protocols and the detailed patient characteristics have been reported in the literature [14].

### RNA sequencing variants calling

The preprocessing of the Fastq files was conducted using FastQC version 0.11.9 [15]. Trim galore, a wrapper around Cutadapt version 3.7 and FastQC, was used for the adapter trimming and to do further quality assessment of the raw file [16]. The splice-aware genome aligner STAR was used to align adapter-trimmed single-end reads to the human reference genome (hg38) [17]. The alignment post-processing was then conducted using the Picard tool (https://broadinstitute.github.io/picard/) with the “Picard markDuplicates” command to mark duplicate reads. Splitting reads that contain Ns in their cigar string was done using Genome Analysis Tool Kit 4 (GATK4) [18] using the “GATK4 SplitNCigarReads” function. The GATK4 Base Quality Recalibration (BSQR) was then done on the aligned reads. Calling single nucleotide polymorphisms (SNPs) and insertions and deletions (indels) via local re-assembly of haplotypes was conducted using the “GATK4 HaplotypeCaller” function. The identified variants were further filtered using the “GATK4 VariantFiltration” command. Finally, the overall quality of the alignment and the data, in general, was assessed using MultiQC software [19]. The reported variants were then annotated to study their effects on proteins and genes using the variant effect predictor (VEP) tool [20], using “homo_sapiens” as the target organism. The VEP was also used to identify the variant genes later used for the gene enrichment analysis using Clusterprofile [21], a Bioconductor package. These analyses were conducted using the rnavar (https://github.com/nf-core/rnavar), which is part of the nf-core pipelines [22]. The annotation of the identified SNPs was conducted using the SNPsnap tool [23]. Downstream data analysis and visualization were conducted in the R programming language.

## Results

This study assessed the expressed RNA variants profile of the critical (individuals in the Intensive Care Unit under ventilation) and non-critical (individuals admitted in the non-critical care wards) following SARS-CoV-2 infection. Characterizing the expressed RNA variants from the patient mentioned above cohorts will help us gain more insight into what constitutes SARS-CoV-2 pathogenesis in our population.

### Critical and non-critical SARS-CoV-2 patients clustered according to the disease condition

**Fig. 1:**
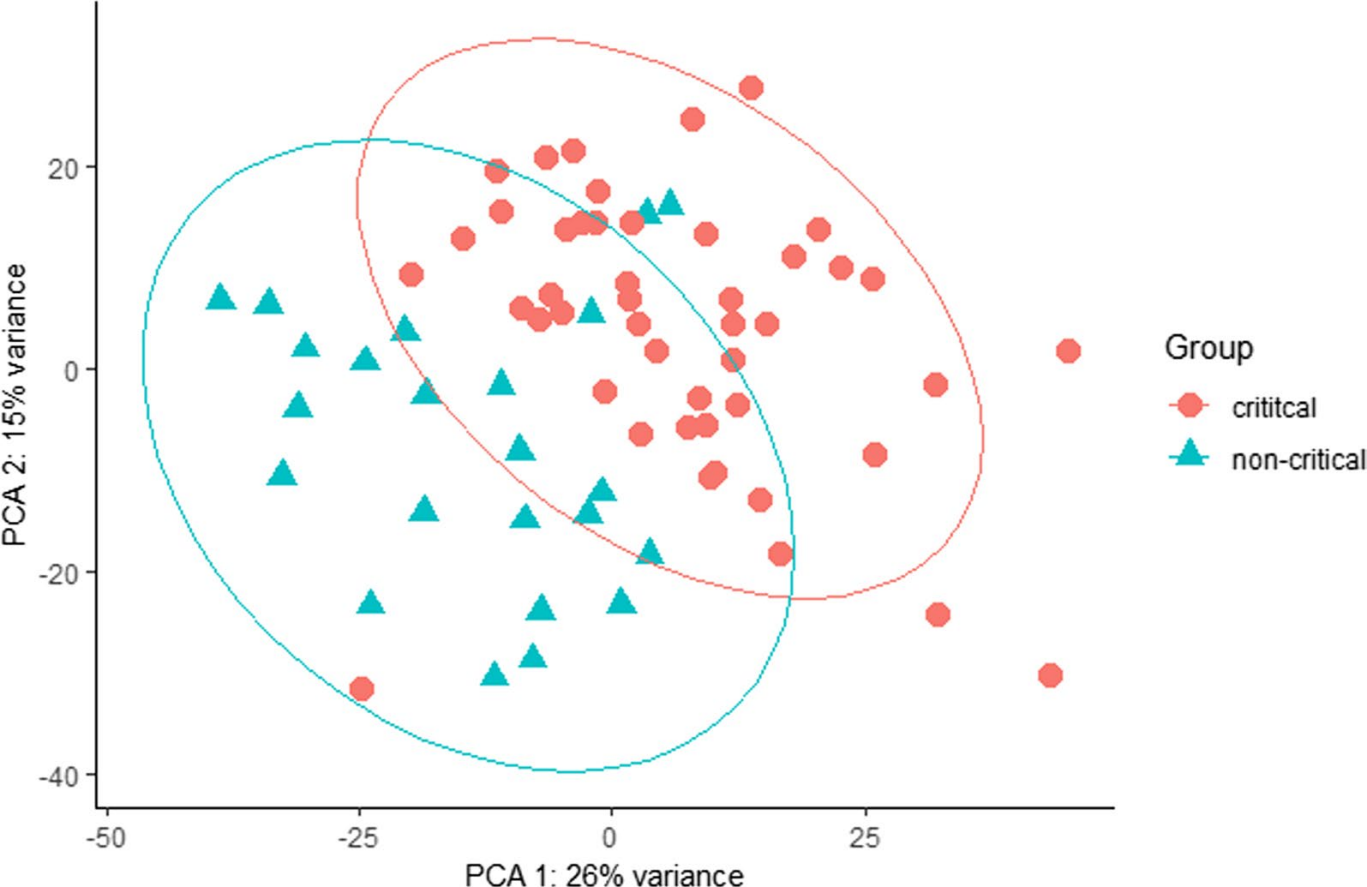
Principal component analysis (PCA) showing segregation of critical and non-critical SARS-CoV-2-infected individuals. The “red” dots represent critical patients, and the “green” dots represent non-critical patients. The contribution of each PCA is shown on the *X*- and *Y*-axis where PCA 1 contributed 26% of the variance and PCA 2 contributed 15% of the total variance in the data

A recent study using multi-omics approaches such as proteomics, transcriptomes phosphoproteome, and ubiquitinome demonstrated that SARS-CoV-2 infections cause perturbations of the host upon infection at different omics levels [24]. Following SARS-CoV-2 infections in human hosts, it has been demonstrated that it affects different body sites, such as lung injuries [5]. To this end, I assessed the difference between critical and non-critical patient cohorts following SARS-CoV-2 infection. According to our data, there is a difference in the RNA expressed variants in critical and non-critical SARS-CoV-2-infected individuals in Fig. 1. I observed non-critical individuals clustering with the critical patients' cohorts, which could be due to the misclassification of the patients in a health facility. The overlap in patient clustering could also be attributed to the host’s different responses, which is expected in a population.

### Expressed variants demonstrated differences in abundance in critical and non-critical conditions

**Fig. 2:**
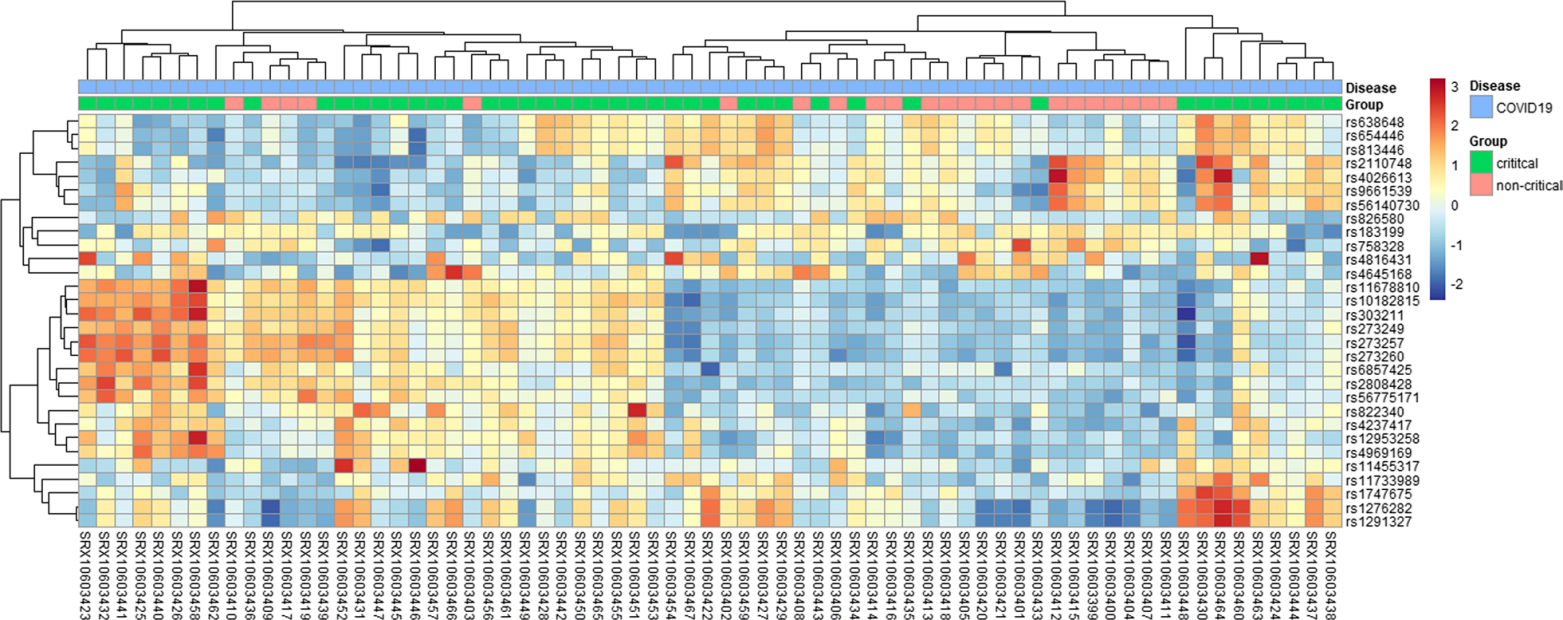
Heatmap showing the relative abundance of the top 30 most abundant single nucleotide polymorphisms in critical and non-critical SARS-CoV-2-infected individuals. The samples were both collected from individuals infected with SAR-CoV-2. In the legend, the “green” color denotes the critically ill individuals, and “red” denotes the non-critical individuals. The color scale ranges from red (more abundant) to blue (less abundant) variant types in a given patient cohort

The relative abundance of the expressed RNA variants in the critical and non-critical patients is assessed in Fig. 2. Heterogeneous clustering of patient cohorts was observed where some critical and non-critical patient cohorts clustered together. This is expected since individuals do not mount the same response to the infecting SARS-CoV-2 [1]. The critical and non-critical patient cohorts displayed a considerable difference in the expression of RNA variants, where variants such as rs11678810 and rs10182815 were more abundant in critical and less abundant patient cohorts in Fig. 2. This analysis shows some similarity in the expressed variants that are more abundant in both the patients' cohorts under our consideration. I also observe a clear difference in the expressed variants in Fig. 2.

### Critical and non-critical SARS-CoV-2 patients are characterized by a unique set of expressed RNA variants

**Fig. 3:**
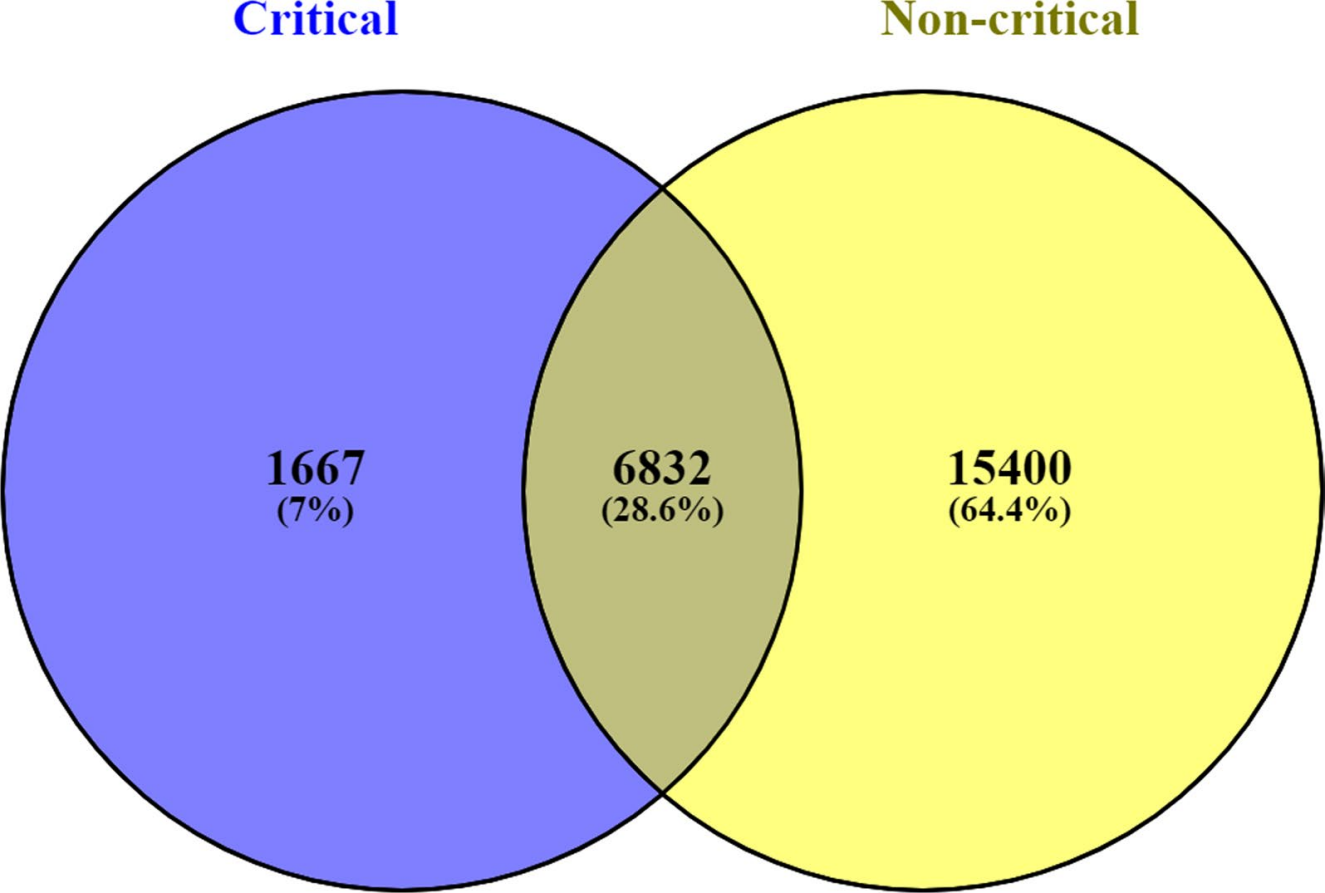
Venn diagram showing variants overlap between critical and non-critical patients’ cohorts. The “blue” color represents critical patients, and the “yellow” color represents non-critical patients. The critical patients had 1667 (7%) of the total unique variants, while non-critical individuals had 15,400 (64.4%) unique variants. There were 6832 (28.6%) common variants between the critical and non-critical patient cohorts

Recent research has shown a broad spectrum of SARS-CoV-2 manifestation in different patient cohorts [25]. The clinical manifestations of SARS-CoV-2 range from mild-to-critical conditions, and there has not been a universal explanation for these clinical observations [25]. In addition, SARS-CoV-2 has been demonstrated to affect different body organs, including the skin and kidney where it causes acute kidney injury, liver, and the gastrointestinal tract [1]. In this analysis, we compared the expressed RNA variants from the two SARS-CoV-2-infected patients cohorts in Fig. 3. Our analysis demonstrates that critical and non-critical SARS-CoV-2 conditions are characterized by different single nucleotide polymorphisms (SNPs) in Fig. 3. There were 6832 (28.6%) common SNPs between critical and non-critical patient cohorts, as shown in Fig. 3. Interestingly, the non-critical patients had 15,400 (64.4%) unique SNPs, while the critical patient cohort had 1667 (7%) unique SNPs in Fig. 3.

### Expressed gene variants are enriched in both innate and cellular immune responses in critical and non-critical patient cohorts

**Fig. 4:**
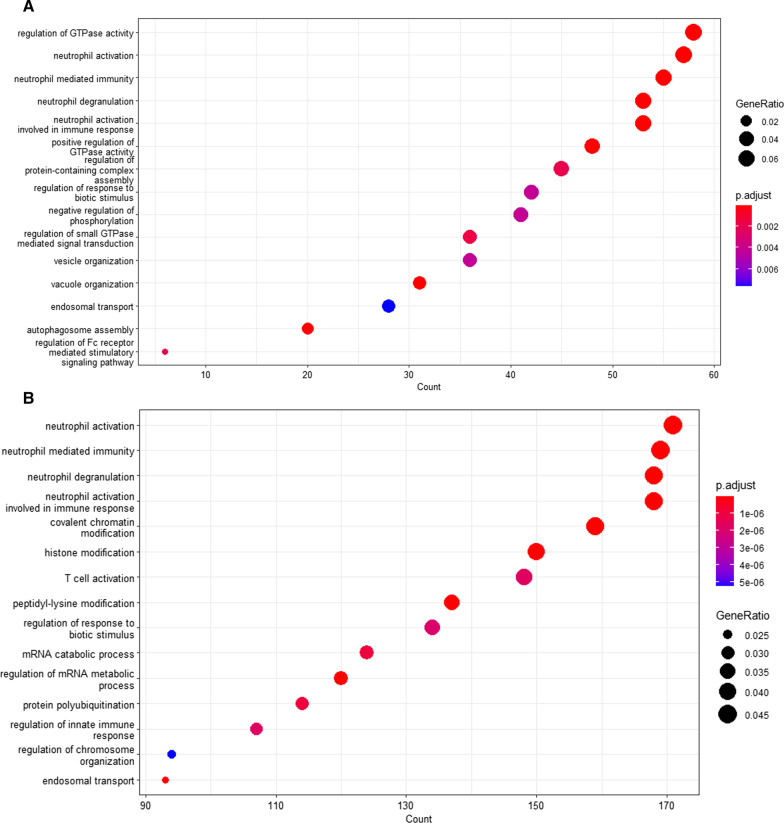
Dot plot showing the top 15 enriched pathways. **A** Enriched pathways in critical patient cohorts, and **B** in non-critical patients' cohorts. The color scale denotes the significance level, with “red” being more significant pathways and “blue” being downregulated pathways. The gene ratio shows the percentage of differentially expressed variants in each gene ontology term

The enriched pathways were investigated following the variants annotations with the variant effect predictor tool. The expressed gene variants in critical and non-critical patients were enriched in both innate and cellular immune responses. In the critical patient cohort, “regulation of GTPase activity,” “neutrophil activation,” “neutrophil-mediated immunity,” “neutrophil degranulation,” and “neutrophil activation involved in immune response” are significantly upregulated in Fig. 4A. In addition to the GTPase mentioned above activity, our analysis also reveals “positive regulation of GTPase activity” and “regulation of small GTPase mediated signal transduction” in the critical patient cohort in Fig. 4A. The “negative regulation of phosphorylation” was also significant in the critical patient cohort, a finding which was consistent with the Bouhaddou et al. [26] finding. In the non-critical patient cohort, the most enriched pathways were: “neutrophil activation,” “neutrophil-mediated immunity,” “neutrophil degranulation,” and “neutrophil activation involved in immune response” in Fig. 4B. Interestingly, the “T cell activation” was significantly enriched in the non-critical patient cohort. Our analysis reveals that the expressed RNA variants in critical and non-critical SARS-CoV-2-infected individuals are mostly enriched in the innate immune response.

## Discussion

Understanding the broad spectrum of SARS-CoV-2 clinical manifestations is yet to be unraveled. Depending on the host immune dynamics, the clinical presentation of host post-SARS-CoV-2 infection can range from asymptomatic to symptomatic [27]. The symptomatic clinical manifestation of SARS-CoV-2 infection ranges from mild, moderate, and severe disease [27]. Since SARS-CoV-2 is a novel virus, the answer to these questions has not been presented and remains an active research area. Previous studies have demonstrated that the muscle pains that characterize SARS-CoV-2 infection in humans are caused by the cytokine storm [28]. The infected individuals' primary cytokine source is the infected macrophages and the lung epithelial cells [28]. Our analysis demonstrated that different profiles of expressed RNA variants characterize critical and non-critically ill SARS-CoV-2-infected individuals. Three non-critical patients clustered together with the critical patients, which can be attributed to the misclassification of the patients or the worsening of the patient condition, which the sample collectors did not notice at the time of sample collection. In general, our data indicate that the critical and non-critical SARS-CoV-2-infected individuals require different management because they display considerable differences when clustered in space [29, 30].

The expressed RNA variants significantly clustered in critical and non-critical patient cohorts. The clustering of these patient groups shows some intrinsic dynamics at the patient cohort level that is specific to SARS-CoV-2 severity [25] as demonstrated in our data. We also observed overlap in patient clustering in the critical and non-critical patient cohorts, which could be attributed to the heterogeneity in immune response, an observation expected in a population since the individuals will not respond uniformly to the infection [31]. We opine that these uniquely abundant expressed variants can be used as molecular markers to classify patients in health facilities to ensure optimal management of SARS-CoV-2 infections. Our data also demonstrate that critical and non-critical showed similarities and differences in the expressed gene variants. This is interesting because this information can be used to understand our population's broad spectrum of SARS-CoV-2 infection.

SARS-CoV-2 has developed the ability to switch on and off the innate and cellular immune response, negatively affecting its pathology [32]. The innate immune response is the first line of defense following pathogen infection. In a study by Cheemarla et al. [33], they demonstrated that innate immune response restricts the initial replication of SARS-CoV-2 following infection. Interestingly, the study demonstrated that the expressed gene variants are enriched in the innate immune response, specifically the neutrophil-mediated immune response. The identities of the expressed stand a chance in adding another layer of information in developing therapeutics and vaccines to help control the SARS-CoV-2 virus spread. The signals of cellular immune response in the non-critical patient cohort were also identified as an indication that the two cohorts are characterized by unique and similar immune responses post-SARS-CoV-2 infection.

SARS-CoV-2 infection is not a binary outcome; its manifestation can range from asymptomatic to mild, severe, and critical [34, 35]. The differential clinical manipulation of SARS-CoV-2 infections provides hitherto undiscovered pathogenesis information, which can be attributed to the difference in the expressed variants, as shown in this study. The analysis reveals that the two patient cohorts: critical and non-critically ill individuals, are characterized by different pathophysiology as demonstrated by the enriched pathways in critical and non-critical patients. The overlap in the enriched pathways is also acknowledged, meaning that our patient cohorts have similarities and differences in response to viral infection. In the literature, it has been demonstrated that the human body organs display differential expression of ACE-2 proteins [36], which also explains the effects SARS-CoV-2 has on different tissue types. Other factors such as age, gender, and host immune system response also play a key role in the pathophysiology of SARS-CoV-2 in the human host [37]. In young comorbidity-free individuals, there are different pathophysiologies, which indicates that the management of these individuals needs to be done based on their disease conditions.

The analysis revealed interesting patterns in the enrichment pathways in critical and non-critical SARS-CoV-2 individuals. The “regulation of the GTPase activity” was the most significantly enriched pathway in critical patients, while in non-critical individuals, “neutrophil activation” was the most significantly enriched pathway. Previous studies have shown that type 1 interferon helps in controlling viral infection in humans through the induction of interferon-stimulated genes, which plays a critical role in controlling viral replication [38]. The infected individuals with viruses express more myxovirus (MX) resistance genes that mainly encode GTPases, which are a very important enzyme responsible for antiviral response [39]. The critically ill individuals encode more variants derived from the MX genes, enabling them to encode more GTPase genes, allowing their immune system to control and fight the viral infection. The innate and T cell immune response [1] is key in controlling viral replication in non-critical patients. The two cohorts differ greatly in the enriched pathways, indicating that their management should be planned differently. The enrichment analysis of the expressed RNA variants in our data demonstrates that the GTPase pathway could be a potential drug target pathway [40]. This information can provide pointers in the drug discovery and development space. A deeper understanding of what constitutes this manifestation needs further investigation.

## Conclusion

The analysis demonstrates that unique sets of expressed RNA variants characterize the critical and non-critical SARS-CoV-2-infected individuals. The findings in this study move us closer to understanding what constitutes different SARS-CoV-2 manifestations in our communities. The different sets of the expressed RNA variants can be used in the identification of critical and non-critical cases of SARS-CoV-2 infections in our health facilities, hence improving the management of SARS-CoV-2.

## Data Availability

The bulk RNA data set used in this study is available on GEO with the accession numbers GSE172114.

## Abbreviations

SARS-CoV-2: Severe acute respiratory syndrome coronavirus *2*
GEO: Gene Expression Omnibus
STAR: Spliced transcripts *alignment* to a reference
RNA: Ribonucleic acid
ACE2: Angiotensin-converting enzyme 2
AKI: Acute kidney injury
VOC: Variants of concern
VOI: Variants of interest
VBM: Variants being monitored
GATK4: Genome Analysis Toolkit 4
VEP: Variant effect predictor
SNPs: Single nucleotide polymorphisms
PCA: Principal component analysis
